# The effects of chitosan supplementation on anthropometric indicators of obesity, lipid and glycemic profiles, and appetite-regulated hormones in adolescents with overweight or obesity: a randomized, double-blind clinical trial

**DOI:** 10.1186/s12887-022-03590-x

**Published:** 2022-09-05

**Authors:** Somaye Fatahi, Ali Akbar Sayyari, Masoud Salehi, Majid Safa, Mohammadhassan Sohouli, Farzad Shidfar, Heitor O. Santos

**Affiliations:** 1 Department of Nutrition, School of Public Health, Iran University of Medical Sciences, Tehran, Iran; 2 Pediatric Gastroenterology, Hepatology, and Nutrition Research Center, Research Institute for Children’s Health, Shahid Beheshti University of Medical Sciences, Tehran, Iran; 3 Department of Biostatistics, School of Public Health, Iran University of Medical Sciences, Tehran, Iran; 4 Department of Hematology and Blood Banking, Faculty of Allied Medicine, Iran University of Medical Sciences, Tehran, Iran; 5 Student Research Committee, Department of Clinical Nutrition and Dietetics, Faculty of Nutrition and Food Technology, Shahid Beheshti University of Medical Sciences, Tehran, Iran; 6 School of Medicine, Federal University of Uberlandia (UFU), Uberlandia, Minas Gerais Brazil

**Keywords:** Chitosan, Obesity, Lipids, Appetite, Adolescents

## Abstract

**Background:**

Chitosan is one of dietary fiber that has received great attention in improving obesity-related markers, but little is known on its effects on adolescents.

**Objectives:**

To analyze the effects of chitosan supplementation on obesity-related cardiometabolic markers and appetite-related hormones in adolescents with overweight or obesity.

**Methods and analysis:**

A randomized clinical trial was performed on 64 adolescents with overweight and obesity, who were randomly allocated to receive chitosan supplementation (*n* = 32) or placebo as control (n = 32) for 12 weeks. Anthropometric measures, lipid and glycemic profiles, and appetite-related hormones were examined.

**Results:**

Sixty-one participants completed study (chitosa*n* = 31, placebo = 30). Chitosan supplementation significantly improved anthropometric indicators of obesity (body weight: − 3.58 ± 2.17 kg, waist circumference: − 5.00 ± 3.11 cm, and body mass index: − 1.61 ± 0.99 kg/m^2^ and − 0.28 ± 0.19 Z-score), lipid (triglycerides: − 5.67 ± 9.24, total cholesterol: − 14.12 ± 13.34, LDL-C: − 7.18 ± 10.16, and HDL-C: 1.83 ± 4.64 mg/dL) and glycemic markers (insulin: − 5.51 ± 7.52 μIU/mL, fasting blood glucose: − 5.77 ± 6.93 mg/dL, and homeostasis model assessment of insulin resistance: − 0.24 ± 0.44), and appetite-related hormones (adiponectin: 1.69 ± 2.13 ng/dL, leptin − 19.40 ± 16.89, and neuropeptide Y: − 41.96 ± 79.34 ng/dL). When compared with the placebo group, chitosan supplementation had greater improvement in body weight, body mass index (kg/m^2^ and Z-score), waist circumference, as well as insulin, adiponectin, and leptin levels. Differences were significant according to *P*-value < 0.05.

**Conclusion:**

Chitosan supplementation can improve cardiometabolic parameters (anthropometric indicators of obesity and lipid and glycemic markers) and appetite-related hormones (adiponectin, leptin, and NPY) in adolescents with overweight or obesity.

## Introduction

Adolescent obesity has emerged as a serious health issue worldwide [1]. Obesity in children and adolescence is often complicated primarily by an early association with a range of other noncommunicable diseases [2–4]. There is a close link between childhood and adult obesity, so much so that a recent systematic review stated that children with obesity had a five times greater risk of having obesity in the adulthood compared with children with normal-weight [5]. Childhood obesity often extends into adulthood, in which approximately 80% of children with obesity have obesity in the adulthood [6].

Lifestyle-based interventions (diet, exercise, and behavioral therapy) together with medications are the most traditional treatments for obesity, leading to reduced caloric intake and increased energy expenditure [7–9]. Nutrition-based therapies associated with a drastic reduction in energy and nutrient intake, however, may be less effective in children and adolescents in virtue of lack of adherence, hence with ensuing weight gain as well as micronutrient deficit [10–14]. Instead of the concept of food restriction, an increment of functional foodstuffs or supplements may be conceivable in this setting. For example, high-fiber foods or fiber supplementation can elicit several improvements in cardiometabolic parameters in different populations [15, 16]. It is no wonder that proper fiber intake is recognizably associated with reduced risk of obesity and seems to be viable in managing the pediatric population [17, 18].

Among the types of fiber, chitosan is one of them that has received great attention in preventing fat absorption [19]. It is a cationic polysaccharide derived from crustacean cuticles such as shrimp and lobster, or fungal wall by distillation (hydrolysis of N-acetyl-D-glucosamine units) of chitin biopolymer [20]. In animal models, chitosan upregulates the hepatic low-density lipoprotein (LDL) receptor mRNA expression and increases the excretion of fecal bile acids, thus decreasing total cholesterol and low-density lipoprotein cholesterol (LDL-C) levels [21]. Additionally, chitosan can increase leptin concentrations and reduce expression of the neuropeptide Y (NPY) gene in the jejunal region, which is an potent satiety and an appetite-stimulating hormone, respectively, favoring therefore weight loss [22, 23].

Despite the myriad metabolic effects of chitosan, further human research is deemed of paramount importance primarily in the pediatric population, including teenagers. In light of this wisdom, this randomized clinical trial (RCT) was conducted in order to analyze the effects of chitosan supplementation on appetite-related hormones, anthropometric indicators of obesity, and lipid and glycemic profiles in adolescents with overweight or obesity.

## Material & Methods

### Participants

**Fig. 1:**
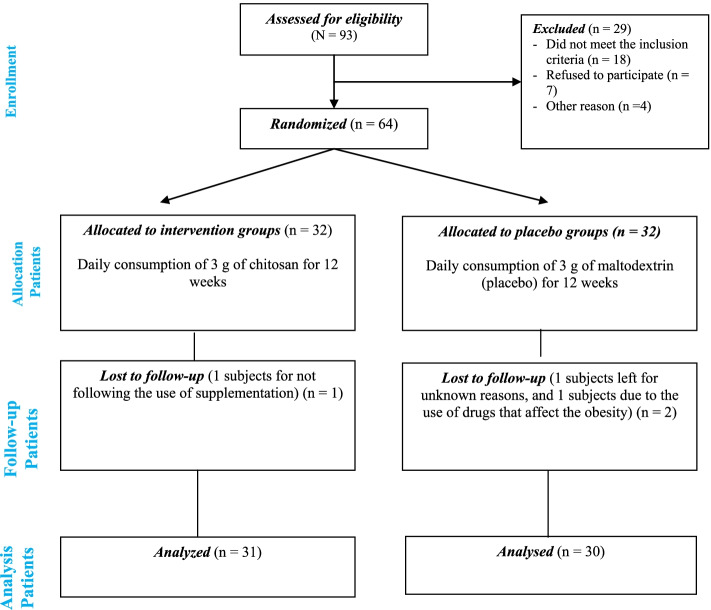
Consort flow diagram for the trial

A double-blind RCT was conducted during 2021–2022, involving adolescents with overweight or obesity referred to the Obesity Clinic of the Mofid Children’s Hospital, Tehran, Iran, who were selected based on inclusion and exclusion criteria. The ethics committee of the Iran University of Medical Sciences approved the study. Moreover, this clinical trial was registered on the Iranian Registry of Clinical Trials (www.irct.ir) website (IRCT20091114002709N57; registration date: 2021-06-20). The flow chart of the study design and the schedule of the project are shown in Fig. 1.

### Inclusion and exclusion criteria

Inclusion criteria included the following items: 1) Willingness to cooperate and sign the informed consent form after full knowledge of the objectives and method of the study; 2) Adolescent girls and boys with overweight and obesity, aged 10 to 19 years; 3) body mass index (BMI) Z-score higher than 1 and less than 3 based on age and sex (according to the definition of World Health Organization (WHO) [24, 25]). Also, adolescents were not included in the study if they meet the following criteria: 1) Use of probiotic supplements, prebiotics, symbiotics or any foods fortified with these supplements during the last 3 months; 2) Use of any antibiotics for 3 months before the study; 3) History of type 1 or 2 diabetes, cardiovascular, hepatic, gastrointestinal (celiac disease, irritable bowel syndrome, and inflammatory bowel disease) or renal diseases and metabolic disorders including maple syrup urine disease and phenylketonuria, urea cycle disorders; 4) History of gastrointestinal surgery; 5) Use medications or supplements that affect appetite, weight, or metabolism at least 3 months before the study (such as medications that affect carbohydrate, protein, or fat metabolism, and also medications that reduce or increase appetite or food intake, including herbal supplements); 6) Adherence to weight loss diet or any type of heavy exercise program during the last 6 months; 7) Pregnant and lactating adolescents; 8) Smoking (more than one cigarette in the last week or more than 200 cigarettes in Lifespan); 9) Having any allergy to chitosan or crabs and shrimp. We also excluded those who with any acute illness, the occurrence of any accident that affects a person’s health, the use of antibiotics during the study, failure to follow the supplement based on personal reasons or other reasons, and migration. Furthermore, the admission rate of patients after the intervention period was calculated using the following formula, and patients whose admission rate is less than 80% were excluded from the study. Acceptance rate = number of packages received at the beginning of the study/number of packages consumed at the end of the study * 100.

### Sample size calculation

Given the absence of a study that investigated the effect of chitosan on weight loss in children and adolescents with overweight or obesity, we used a method of Reinehr et al. in order to calculate the sample size by considering the BMI Z-score as the primary outcome, as these authors examined the effects of prebiotics on overweight and overweight children. In this way, considering the difference of 0.066 units in the mean BMI Z-score at the end of 12 weeks of intervention, assuming S1 = 0.07 and S2 = 0.09, and with the type I of error probability level of 5% (α = 0.05) and the type II error probability level of is 20% (β = 0.20, power = 80%), the number of samples was calculated based on the sample size formula below 24 participants in each group. Assuming 30% of the possible loss, 32 participants in each group and a total of 64 participants were included in our study.

### Study design and intervention

In this randomized double-blind randomized clinical trial with 12 weeks of intervention, 64 adolescents with overweight or obesity who meet the inclusion criteria were randomly divided into two groups receiving chitosan supplement and placebo (maltodextrin). The appropriate amount of chitosan supplementation in most studies is approximately 3 g/d [26–28]. Since no toxicity of this substance has been reported to mammals in the FDA [29], the participants received 1.5 g (Twice a day a total of 3 g) of chitosan powder (intervention group) or maltodextrin (placebo group) daily 30 minutes to 1 hour before lunch and dinner for 12 weeks. Standard fruit flavorings were added to these supplements, taking into account the possibility of individuals not consuming raw chitosan powder. Parents were advised to add the recommended amount of powder for each person to 250 ml of water. The supplements were provided by Karen Pharmaceuticals and Vital-Food Supplements Company. The powders were given to the parents at the beginning of the study and at the end of the fourth eighth week, and they are asked to bring empty packages of cans at the end of the fourth, eighth and twelfth weeks to check the acceptance rate of supplements.

At baseline, all study participants received recommendations for gradual weight loss (0.5 to 1 kg per month). According to age, sex, height, and BMI Z-score, the energy consumption was calculated according to the formulas proposed in Krauss’s book and a slight reduction of 200 kcal per person is considered [30]. The caloric distribution of the diet was estimated at 30% fat (7% saturation), 50% carbohydrate, and 20% as protein and the maximum amount of cholesterol and 300 mg per day. Dietary recommendations were the same for both groups, and both groups are asked not to consume fortified sources of probiotics, symbiotics, or prebiotics or supplements during this study.

### Randomization and allocation

To ensure the uniform distribution of the main variables can have a great impact on the results (BMI Z-score and gender) in two groups, we used random allocation by Stratified Randomization and Permuted block randomization method. Based on the sample size of present study (64 subjects), we produced the double block and quadruple block using the online site (www.sealedenvelope.com). At the beginning of the study, sets of packages containing chitosan powder were prepared by someone other than the researcher due to the double-blindness of the study, and the placebo was similar in appearance to chitosan powder. All of the researchers from the allocation of participants in each of the groups (intervention and control group) until the end of the intervention, were not know the groups whereby the patients were randomized. In order to apply concealment in the randomization process, we used unique codes which generated by the company receiving the supplements and placebos on the medicine boxes. So, none of the participants and researchers know which of the two groups received the supplement or placebo with this method. Upon each person entering the study, based on the sequence generated, the medicine boxes in which the code is recorded was assigned to the participants.

### Evaluation of personal information

At the beginning of the study, personal information including name, age, sex, dietary supplements, and history other diseases were completed using the face-to-face interview technique (person or parents). Maturity status is determined by a trained individual using Marshall and Tanner tables [31].

### Anthropometric and physical activity measurements

Anthropometric variables were measured before and at the end of the study. Adolescents’ height and weight were measured with minimal clothing and without shoes. The weight of all subjects was measured twice with the Seca digital scale (made in Germany) with an accuracy of 0.01 kg. The height of the participants in the study was recorded standing using a tape measure, without shoes and with an accuracy of 0.5 cm at the beginning and end of the study; measures were collected twice each time and their average was recorded). BMI was determined as weight in kilograms divided by height in meters squared. Patients’ waist circumference was measured using a non-elastic tape measure with a maximum error of 0.5 cm, considering the middle half of the body below the ribs of the chest.

The BMI Z-score, also known as the standard deviation for BMI score, is a measure of relative weight and height that is set for age and gender in a reference standard. These scores are considered more suitable for determining longitudinal changes in body weight and obesity, and are also a superior criterion for comparing the mean values of the group [32]. Therefore, BMI Z-score was used to assess changes in body weight in participants. The level of physical activity at the beginning and end of the study was assessed by the International Physical Activity Questionnaire (IPAQ) in Persian. The amount of physical activity was calculated as small continuous data taking into account the coefficients related to the activity, and recorded as Met-min/week. The Met coefficient for walking is 3.3, for moderate activity is 4, for heavy activity is 8, which is multiplied by the duration of the activity in minutes and the number of days in the activity week, and its sum as the amount of physical activity in the week is set [33].

### Biochemical measurements

At the beginning of the study and at the end of the twelfth week, after 12 to 14 hours of fasting, 5 cc of a venous blood sample was taken from the patients while sitting on a chair. These samples were centrifuged at room temperature for 10 minutes at 3700 rpm to separate their serum. The isolated serum was placed in 1.5 cc microtubules to measure biochemical factors was stored in a freezer at − 80 °C until testing.

Serum total cholesterol and triglyceride (TG) levels were measured using Pars Azmoon commercial kits (Tehran, Iran) by a biochemistry autoanalyzer, and serum high-density lipoprotein cholesterol (HDL-C) were measured after deposition of apolipoprotein B-containing lipoproteins with phosphotungstic acid solution. In cases where the triglyceride level was less than 400 mg/ml, serum LDL-C levels were calculated using the Friedewald Equation [34]: LDL-C = total cholesterol -TG/6 - (HDL-C). In other cases, commercial kits were used as a surrogate measure.

Pars Azmoon commercial kits (Tehran, Iran) were used to measure fasting blood glucose (FBG) by a biochemistry autoanalyzer and serum insulin levels by the immuno-turbidimetry method. Homeostatic model assessment of insulin resistance (HOMA-IR) was calculated as fasting insulin (mU/L) × FBG (mmol/L)∕405. Serum NPY was assessed using an ELISA kit (Crystal Day Biotech Co, Shanghai, China). Leptin and adiponectin were measured by using an ELISA kit (Mediagnost Co, Germany).

### Dietary assessment

Evaluation of dietary intake at the beginning and end of the study and each time using a 24-hour dietary recall questionnaire of 3 days (2 normal days and 1 day off) was done interviewing the adolescents or parents. Related data, including energy intake, macronutrients, and some micronutrients were determined by Nutritionist 4 software.

### Statistical analysis

SPSS-24 software (IBM Corp. IBM SPSS Statistics for Windows, Armonk, NY) was used to obtain statistical analyses. Quantitative variables were reported as mean (standard deviation) and qualitative variables were reported as numbers (percentage). Because the data was not normal, Mann-Whitney test were used to compare the results between baseline and end of the intervention between groups. As well as Wilcoxon test, were used to analyze within-group data. ANCOVA test was used to estimate any differences in treatment group at the end of trial with adjusting for covariates. Also, Chi-square test were used to compare qualitative factors. Significant levels for all tests were considered as *P*-value < 0.05.

## Results

### Characteristics of the participants

From 93 pediatric patients with overweight or obesity eligible for inclusion, 64 were selected and 61 participants completed study and entered the final analysis (31 in the intervention group and 30 in the placebo/maltodextrin group) (Fig. 1).

The baseline characteristics of the participants are presented in Table 1. The mean age of the participants was 13.51 years in the intervention group and 13.12 years in the control group. The mean and standard deviation of BMI (Z-score) of the participants group was 1.52 (0.26) in the chitosan and 1.67(0.32) in the placebo group which was not statistically significant. Also, there was no significant difference between the two groups in terms of age, gender, waist-circumference, physical activity and multivitamin supplement consumption distribution (*P* = 0.891 and *P* = 0.893, *P* = 0.196, *P* = 0.778 and *P* = 0.704, respectively).

**Table 1:** Baseline characteristics of participants

Variables	Groups, mean (SD)		***P***-value^**a**^
	Chitosan (*n* = 31)	Control (*n* = 30)	
**Age(y)**	13.51 (2.15)	13.12 (2.02)	0.891
**Female (n, %)**	14 (48.4)	15 (46.7)	0.893
**Height (cm)**	148.74 (9.15)	152.21 (8.48)	0.130
**Weight (kg)**	56.12 (7.20)	57.67 (9.34)	0.702
**BMI (kg/m**^**2**^**)**	25.31 (1.79)	24.71 (1.86)	0.098
**BMI (Z-score)**	1.52 (0.26)	1.67 (0.32)	0.064
**Waist-circumference (cm)**	88.48 (15.15)	93.40 (16.39)	0.196
**Physical Activity (met.h/wk)**	472.14 (225.33)	528.28 (327.04)	0.778
**Multivitamin use (n, %)**	6 (19.4)	7 (23.3)	0.704

*BMI* Body mass index, *DBP* Diastolic Blood Pressure, *SBP* Systolic Blood Pressure, *WC* Waist circumference

^a^ Data obtained from Mann-Whitney Test for continuous variables and Chi-square for categorical variables

Dietary intake is indicated in Table 2. Based on the findings of the 24-h dietary recall questionnaire and comparing the beginning with the end of the study, the analysis of findings shows that although intake of energy (*P* = 0.039), protein (*P* = 0.031), total fat (*P* = 0.018), saturated fat (*P* < 0.001), polyunsaturated fat Saturation (*P* = 0.015), vitamin E (*P* = 0.036) and zinc (*P* = 0.001) showed a significant decrease after the intervention in the chitosan supplement group, however, these changes were not significant between the two groups. Regarding the intake of other macronutrients and micronutrients, no statistically significant difference was observed between the two groups before and after the intervention.

**Table 2:** Energy, macronutrient, and micronutrients intake at baseline and at the end of study

	Chitosan			Placebo			***P***-value^**b**^
	Baseline	After	*P*-value^a^	Baseline	After	*P*-value^a^	
**Energy (kcal/d)**	2181.43 (204.41)	1896.10 (233.05)	**0.021**	1961.79 (244.82)	1887.78 (311.85)	0.171	0.906
**Carbohydrate (g/d)**	276.95 (67.57)	271.35 (71.01)	0.754	262.18 (78.22)	262.58 (80.90)	0.177	0.433
**Protein (g/d)**	72.51 (19.99)	69.65 (21.96)	**0.031**	71.50 (14.40)	69.78 (15.31)	0.069	0.812
**Fat (g/d)**	59.43 (13.94)	55.65 (16.43)	**0.018**	55.17 (19.88)	57.94 (17.75)	0.254	0.812
**SFA (g/d)**	20.20 (6.11)	16.67 (5.49)	**< 0.001**	18.69 (4.97)	18.41 (5.66)	0.854	0.624
**MUFA (g/d)**	19.55 (6.77)	17.49 (6.79)	0.053	16.87 (6.96)	17.33 (6.50)	0.430	0.988
**PUFA (g/d)**	20.76 (8.88)	17.58 (8.06)	**0.015**	19.64 (8.60)	20.40 (9.03)	0.629	0.319
**Cholesterol (mg/d)**	182.14 (81.94)	175.85 (66.63)	0.991	183.18 (64.99)	175.46 (67.97)	0.517	0.956
**Fiber (g/d)**	17.92 (6.45)	17.29 (6.88)	0.094	17.74 (8.13)	17.67 (8.50)	0.419	0.891
**Vitamin B12 (mcg/d)**	1.47 (0.90)	1.41 (0.70)	0.621	1.37 (0.74)	1.37 (0.78)	0.894	0.415
**Folate (mcg/d)**	249.88 (104.34)	237.25 (97.72)	0.122	280.98 (132.91)	270.07 (133.81)	**0.035**	0.466
**Magnesium (mg/d)**	207.40 (57.45)	196.33 (56.41)	0.469	200.38 (75.90)	200.37 (64.16)	0.393	0.971
**Vitamin A (RE)**	945.9 (557)	1022.9 (162.8)	0.312	894.5 (490.9)	963.2 (318)	0.447	0.593
**Vitamin E (mg/d)**	10.4 (4.9)	8.3 (6.1)	**0.036**	11.4 (5)	9.8 (7.4)	**0.042**	0.810
**Vitamin C(mg/d)**	90.8 (43)	90.1 (54)	0.701	86.1 (35.3)	97.6 (33.3)	0.670	0.502
**Vitamin D (mcg/d)**	8.7 (6)	9.1 (3.6)	0.481	8.8 (5.3)	9.6 (5.7)	0.268	0.471
**Selenium (mg/d)**	60.9 (39.1)	71.8 (34.1)	0.069	68.2 (30.5)	70.3 (41.7)	0.109	0.482
**Zinc (mg/d)**	12.8 (3.3)	9.7 (2.7)	0.001	9.5 (4.2)	9.3 (4.8)	0.802	0.059

Data are expressed as Mean (SD)

*PUFA* Polyunsaturated fatty acid, *SFA* Saturated fatty acid, *MUFA* Monounsaturated fatty acid

^a^ *P* value for within-group comparison of non-parametric quantitative data using Wilcoxon signed-rank test

^b^ *P* value for between-group comparison of non-parametric quantitative data using Mann–Whitney U-test

Table 3 depicts the mean values of anthropometric indicators of obesity, lipid and glycemic profiles, and appetite-regulated hormones at baseline and after intervention. Chitosan supplementation caused a significant improvement of weight (*P* < 0.001), BMI (*P* < 0.001), BMI Zscore (*P* < 0.001), waist (*P* < 0.001), fasting blood glucose level (*P* < 0.001), HOMA-IR (*P* < 0.001), insulin (*P* = 0.001), total cholesterol (*P* < 0.001), triglyceride (*P* < 0.001), HDL -C (*P* = 0.04), LDL-C (*P* < 0.001), adiponectin (*P* < 0.001), leptin (*P* < 0.001) and neuropeptide Y (*P* = 0.009) in individuals. This decrease for waist circumference, height (*P* = 0.011), HOMA-IR (*P* = 0.001), total cholesterol (*P* < 0.001), triglyceride (*P* = 0.001) and LDL-C. (*P* = 0.01) was also observed in the placebo group. However, comparing the changes of these variables after adjusting the confounders of weight, BMI, WC, TG, TC, physical activity, adiponectin, and energy between the two groups of participants only for weight (*P* < 0.001), BMI (*P* < 0.001), BMI Zscore (*P* < 0.001), waist circumference (*P* < 0.001), insulin (*P* = 0.006), adiponectin (*P* = 0.02) and leptin (*P* = 0.04) was significant following the twelve-week intervention. The change in other risk factors was not different among the groups.

**Table 3:** Anthropometric characteristics and laboratory markers at baseline and at the end of study

	Chitosan				Placebo				***P***-value^**b**^
	Baseline	After	Change	*P*-value^a^	Baseline	After	Change	*P*-value^a^	
**Weight (kg)**	56.12 (7.20)	52.54 (7.07)	−3.58 (2.17)	**< 0.001**	57.67 (9.34)	57.40 (9.45)	−0.27 (1.85)	0.252	**< 0.001**
**BMI (kg/m**^**2**^**)**	25.31 (1.79)	23.70 (1.93)	−1.61 (0.99)	**< 0.001**	24.71 (1.86)	24.60 (2.05)	−0.10 (0.86)	0.102	**< 0.001**
**BMI (Z-score)**	1.52 (0.26)	1.24 (0.36)	−0.28 (0.19)	**< 0.001**	1.67 (0.32)	1.65 (0.35)	−0.02 (0.12)	0.247	**< 0.001**
**WC (cm)**	88.48 (15.15)	83.48 (15.18)	−5.00 (3.11)	**< 0.001**	93.40 (16.39)	92.40 (16.56)	−1.00 (2.37)	**0.011**	**< 0.001**
**FBG (mg/dL)**	96.19 (10.15)	90.41 (8.81)	−5.77 (6.93)	**< 0.001**	95.26 (9.89)	94.40 (9.82)	−0.86 (3.92)	0.237	0.131
**HOMA-IR**	2.37 (0.89)	2.13 (0.70)	−0.24 (0.44)	**< 0.001**	2.89 (1.37)	2.51 (0.93)	−0.38 (0.70)	**0.001**	0.370
**Insulin (μIU/mL)**	23.23 (11.85)	17.71 (8.44)	−5.51 (7.52)	**0.001**	22.11 (14.64)	23.95 (12.49)	1.84 (11.57)	0.206	**0.006**
**TC (mg/dL)**	182.09 (27.40)	167.96 (26.22)	−14.12 (13.34)	**< 0.001**	167.90 (21.92)	163.93 (21.62)	−3.96 (5.39)	**< 0.001**	0.071
**TG (mg/dL)**	149.64 (45.70)	143.96 (40.43)	−5.67 (9.24)	**< 0.001**	125.56 (29.84)	121.70 (28.54)	−3.86 (5.31)	**0.001**	0.847
**HDL-C (mg/dL)**	43.54 (9.58)	45.38 (8.53)	1.83 (4.64)	**0.041**	43.96 (8.34)	44.46 (8.16)	0.50 (1.73)	0.162	0.116
**LDL-C (mg/dL)**	114.43 (33.82)	107.25 (37.06)	−7.18 (10.16)	**< 0.001**	120.60 (26.21)	115.86 (25.29)	−4.73 (10.16)	**0.016**	0.714
**Adiponectin (ng/mL)**	7.55 (4.20)	9.24 (5.42)	1.69 (2.13)	**< 0.001**	10.09 (5.32)	8.93 (3.81)	−1.15 (3.06)	0.075	**0.028**
**Leptin** (**ng/mL)**	47.66 (35.13)	28.25 (20.73)	−19.40 (16.89)	**< 0.001**	50.04 (44.59)	40.90 (33.80)	−9.26 (26.78)	0.382	**0.046**
**NPY** (**ng/mL)**	207.56 (123.54)	165.59 (99.45)	−41.96 (79.34)	**0.009**	228.24 (155.04)	233.62 (155.82)	5.38 (45.99)	0.910	0.278

Data are expressed as Mean (SD)

*Abbreviations*: *BMI* Body mass index, *FBG* Fasting blood glucose, *HDL-C* High density lipoprotein-cholesterol, *HOMA_IR* Homeostatic model assessment-insulin resistance, *LDL-C* Low density lipoprotein-cholesterol, *NPY* Neuropeptide Y, *TC* Total cholesterol, *TG* Triglycerides, *WC* Waist circumference

^a^ *P*-values for comparison of within-group differences by Wilcoxon signed-rank test

^b^ *P* value for between-group comparison using analyses of covariance (ANCOVA), considering baseline values (weight, BMI, WC, TG, TC, physical activity, adiponectin, and energy) as covariate

## Discussion

Due to the fact that chitosan is not fully digested and absorbed in the body, it has been suggested as a factor in improving weight and cardiovascular risk factors [35]. Chitosan and its derivatives are widely distributed in various health stores and pharmacies and are used by the general public, especially adults [36]. However, its use at an early age has not yet been fully explored. For this reason, in this study, we examined the effects of chitosan supplementation in adolescents with overweight or obesity. In general, our study showed that chitosan at 3/g/d for 12 weeks improved all obesity-related cardiometabolic markers (anthropometric indicators of obesity and lipid and glycemic markers) assessed as well as appetite-related hormones (adiponectin, leptin, and NPY). When compared with the placebo group, chitosan supplementation had greater improvement in body weight, BMI (kg/m^2^ and Z-score), waist circumference, as well as insulin, adiponectin, and leptin levels.

Regarding the weight-loss effect, we found a significant intragroup decrease in body weight of ~ 3.6 kg for chitosan supplementation, while body weight did not change in the placebo group. Such a reduction is higher than the overall result of a meta-analysis (14 selected RCTs) addressing adults with overweight or obesity [37], whereby there was a weight loss of ~ 1 kg (WMD 1.01, 95% CI: − 1.67 to − 0.34) for the chitosan group versus placebo. Moreover, we found a mean BMI decrease of 1.6 kg/m^2^ (− 1.54 ± 1.66) and no change in the placebo group, whose result is similar to the overall BMI difference of − 1.27 kg/m^2^ (95% CI − 1.96 to − 0.57) for the between-group results of the meta-analysis.

Despite our similar results with this aforementioned meta-analysis [37], we observed significant improvements in traditional lipid markers (mean decreases of 14 mg/dL for TC, 7 mg/dL for LDL-C and 6 mg/dL for TG, and 2 mg/dL increase for HDL-C levels) that can be deemed clinically modest, whereas the meta-analysis found expressive reductions in TC (− 54 mg/dL), LDL-C (32 mg/dL), and TG (94 mg/dL) levels. The chitosan dosage of our study is within the dosing range of the meta-analysis, in which a mean of 2 g/d (0.34–3.4 g/d) was used for 17 weeks (4–52 weeks); thus, we tested a feasible therapeutic dosage regimen.

Beyond the scientific scrutiny of assessing meta-analyses, it is crucial to consider well-controlled RCTs apart in order to investigate specific conclusions. In contrast to our study, an RCT consisting of middle-aged patients (*n* = 130 at randomization period) with borderline/mild hypercholesterolemia (186–263 ng/dL) did not find lipid-lowering effects of chitosan supplementation (2.4 g/d over 10-month intervention with alternating periods according to the crossover design) [38]. In line with our findings, in an RCT consisting of adults with obesity (*n* = 94), 2.5 g/d of chitosan supplementation for 3 months led to a mean body weight decrease of 3 kg accompanied by reductions in BMI (− 1.20 kg/m^2^), body fat (− 0.98%), visceral fat (− 1.28%), upper abdominal circumference (− 2.17 cm), hip circumference (− 2.07 cm), and waist circumference (− 1.97 cm) compared to placebo. Of note, chitosan was also able to lower HbA1c to less than 6% in those with higher baseline levels [19]. Such a study is of pivotal importance taking into account the relevance of examining the weight-loss effect and related outcomes of chitosan supplementation in patients with obesity.

Concerning the lipid-lowering effects of chitosan, the action on the gastrointestinal tract is the central tenet, where chitosan, due to the cationic nature, binds to negatively charged lipids and thus reduces their absorption, yielding potential to reduce lipid markers and anthropometric indicators of obesity thanks to fecal excretion of fats [39]. The binding of chitosan to fats and bile acids can also be beneficial for various metabolic factors [40]. More specifically, chitosan dissolves in the stomach and afterward binds to intestinal fat through fat emulsions and gel formation, thereby impairing fat absorption [27, 41]. With respect to the anti-diabetic potential, changes in the expression regulation of peroxisome proliferator-activated receptors (PPAR), in the paraventricular nucleus, have been observed in animals supplemented with chitosan [23]. Recognizably, PPAR activation in patients with type 2 diabetes enhances insulin and glucose levels [42].

The benefits in obesity-related markers that we found can be strongly associated with a mean reduction of ~ 200 kcal/d (from 2181.43 ± 204.41 to 1896.10 ± 233.05 kcal/d) in the chitosan group, while the energy intake did not alter in the placebo group. Such a result may be associated with the appetite-suppressing properties of chitosan, as our study provides evidence that chitosan supplementation can modulate appetite-related hormones. We observed a significant increase and decrease in adiponectin and leptin levels, respectively, which reached both within-group and between-group differences. Furthermore, we noted a significant within-group decrease in NPY levels after chitosan supplementation, that, despite the lack of statistical between-group difference, the ~ 42 ng/mL reduction for chitosan supplementation is clinically meaningful, while concentrations tend to increase by ~ 5 ng/mL in the placebo group.

Taken together, many animal studies furnish the role of leptin, adiponectin, and NPY in appetite modulation and systematic effects on obesity. Chitosan administration has anti-obesity and anti-diabetic effects in ob/ob mice, with related improvement in adiponectin resistance and its plasma concentrations, as well as increased expression in adipose tissue of PPAR-gamma, a key regulator of adiponectin production [43]. Serum leptin concentration and its receptor expression in adipose tissue increased in chitosan fed-pigs compared with animals receiving basal diet, and increased expression of NPY in the hypothalamic nuclei and in the jejunum [23]. In addition to the anorexigenic (i.e., appetite suppressant) role of leptin, experimental data also provide a theoretical basis for systemic effects on chitosan administration by increasing the expression of liver leptin receptor b-L (LepRb) and phosphorylation of JAK2 and STAT3 [44], in which chitosan activates the JAK2-STAT3 signaling pathway and hence reduces leptin resistance while partly suppressing adipogenesis.

### Strengths and limitations

The main strength of this study is the RCT design and novelty, given that this study was the first human research that examined the effects of chitosan supplementation on adolescents with overweight or obesity. However, our studies have limitations that serve as perspectives. First, we did not assess body fat, which should preferably be examined by a reliable method. Second, although we measured some appetite-related hormones, we did not assess ghrelin levels or perform appetite questionnaires. Third, we encourage further RCT to assess these markers, as well as crossover acute studies using transabdominal ultrasound to allow the examination of the gastric emptying on meal tests with and without chitosan supplementation. At last, further research is also required to better understand the effects of chitosan on the gut microbiota, as well as on advanced biomarkers of the lipid profile (e.g., lipoprotein (a) and small dense low-density lipoprotein-cholesterol) and inflammatory and oxidative status due to their emerging scientific attention to disease management [45, 46].

## Conclusion

In general, our study showed that chitosan supplementation can improve cardiometabolic parameters (anthropometric indicators of obesity and lipid and glycemic markers) and appetite-related hormones (adiponectin, leptin, and NPY) in adolescents with overweight or obesity. However, the effects must be considered as an adjuvant instead of a magic bullet for the management of obesity. Preferably, such a strategy ought to be planned mainly in combination with a hypocaloric diet and physical exercise supervised by proper heathy professionals.

## Data Availability

The data that support the findings of this study are available from “ Department of Nutrition, School of Public Health, Iran University of Medical Sciences, Tehran, Iran” but restrictions apply to the availability of these data, which were used under license for the current study, and so are not publicly available. Data are however available from the authors upon reasonable request and with permission of “Dr. Farzad Shidfar, Department of Nutrition, School of Public Health, Iran University of Medical Sciences, Tehran, Iran. E-mail: shidfar.f@iums.ac.ir“.
